# Adolescent Healthcare Autonomy: Mature Minors in the Islamic Context

**DOI:** 10.7759/cureus.86162

**Published:** 2025-06-16

**Authors:** Bilal Irfan, Basel Tarab

**Affiliations:** 1 Center for Surgery and Public Health, Brigham and Women's Hospital, Boston, USA; 2 Michigan Alzheimer's Disease Center, University of Michigan, Ann Arbor, USA; 3 Center for Bioethics, Harvard Medical School, Boston, USA

**Keywords:** adolescent autonomy, decision-making process, pediatric autonomy, pediatric ethics, religious ethics

## Abstract

Contemporary statutes typically define adulthood at 18, yet Islamic tradition considers puberty and intellectual discernment as markers of maturity. This discrepancy creates significant ethical and legal dilemmas for adolescent healthcare autonomy, particularly in Muslim contexts. Classical Islamic jurisprudence emphasizes cognitive maturity alongside physical development, aligning with modern secular concepts like the mature minor doctrine. Nevertheless, tensions arise when adolescents deemed religiously mature must navigate healthcare decisions restricted by civil statutes. This editorial advocates for integrating Islamic ethical frameworks with contemporary capacity-oriented policies, suggesting joint jurist-physician assessments as a practical reconciliation. There are proposals sketched for clear, capacity-oriented guidelines that respect both civil mandates and religious values. Interdisciplinary guidelines can harmonize religious recognition of adolescent autonomy with protective civil mandates, ultimately respecting adolescents’ evolving capacities and healthcare rights.

## Editorial

Contemporary statutes of civil law generally fix 18 years as the age of majority, yet puberty has served for centuries as the gateway to religious and legal adulthood in many parts of the world, including within the confines of Islamic law [1]. Muslims currently are estimated to represent a quarter of the world’s population and have the youngest median age among major religious groups, yet the intersection of their varied religious, legal, and social traditions with healthcare is still under-researched. Tensions arise when an individual who is recognized as an adult under religious precepts is still deemed a minor by civil statutes, particularly for pivotal healthcare decisions [2,3]. In most international and national contexts, protective measures mandate that parental or guardian consent guide treatment decisions until 18, regardless of the adolescent’s maturity. However, many Muslim families point to puberty (bulūgh) and intellectual discernment (rushd) as grounds for recognizing full moral accountability earlier than the civil threshold. It is important to recognize that there is a scholarly difference across the Islamic schools of jurisprudence with respect to the threshold for attaining the status of puberty and the criterion for evaluating discernment.

Historically, puberty-based adulthood granted adolescents significant agency across various spheres in Muslim societies, from fulfilling religious obligations to participating in civil contracts. Yet classical jurists also stressed that physical maturity alone did not automatically confer unlimited autonomy. Evidence of sound judgment was invoked at times to ensure that youth had the cognitive ability to manage complex matters such as property, finances, and, potentially by extension, healthcare. Contemporary Islamic bioethical debates similarly invoke notions similar to the Western ethical concept of no harm (lā darar wa lā dirār), asserting an obligation to preserve life and health while also recognizing adolescents’ developing moral agency and decision-making capacity.

Despite such nuance, statutory laws worldwide typically rely on bright-line ages for decision-making. The Convention on the Rights of the Child enshrines 18 as the standard age of adulthood while emphasizing children’s evolving capacities, urging that their views be given due weight with respect to their age and maturity. In practice, many jurisdictions do allow exceptions under a mature minor doctrine when an adolescent can demonstrate adult-level decision-making [4]. Nevertheless, conflicts remain present in high-stakes scenarios, for example, when a physically mature teenager refuses chemotherapy. In such cases, clinicians and courts may override the refusal, invoking the child’s best interests.

Islamic jurisprudence itself may provide a basis for reconciliation. Modern scholarship, if engaging in jurist-physician didactics, may come to see puberty and evidence of sound judgment as dual prerequisites or another negotiated model, paralleling many child-capacity models in secular ethics. Where a Muslim adolescent demonstrates a clear understanding of and reasoning with a given matter, it can align with capacity testing that contemporary healthcare systems already employ. However, statutory codes in many Muslim-majority states have also adopted 18 as the legal age of majority, while some families still regard puberty as decisive. This dualism can produce confusion over consent for healthcare, such as reproductive or end-of-life care, with adolescents caught between religious recognition of adulthood and civil constraints.

A feasible path forward rests on capacity-oriented policies that integrate Islamic and civil frameworks. Such policies could allow an adolescent under 18, recognized as having reached puberty (bāligh), to be formally assessed for maturity by qualified professionals. If deemed capable, consent or refusal might be honored, and jurist-physician bioethical deliberations can ascertain when life-preserving measures render paternalistic intervention ethically necessary. This middle ground may aid in respecting the no-harm principle, upholding child protection mandates, and echoing the mature minor doctrine. Clear guidelines, jointly developed by medical experts, legal authorities, and Islamic jurists, could avert dilemmas where adolescents risk losing their voice in decisions shaping their health and survival.

These nuanced approaches have a global import, as Islam’s puberty-based paradigm shows that numeric age is not the sole determinant of mature decision-making, a perspective that aligns with broader calls to acknowledge adolescents’ evolving capacities. Integrating religious norms and statutory rules need not be inherently contradictory; in fact, both traditions already prioritize the welfare of those in transition from childhood to adulthood. Yet concrete policy reforms, supported by clinical training in capacity assessment and explicit protocols on adolescent involvement, are needed to ensure that Muslim youth worldwide, as well as healthcare providers, can navigate these ethical junctures with clarity and compassion.
